# Reevaluation of Serum Arylesterase Activity in Neurodevelopmental Disorders

**DOI:** 10.3390/antiox10020164

**Published:** 2021-01-22

**Authors:** Ignazio Stefano Piras, Stefano Gabriele, Laura Altieri, Federica Lombardi, Roberto Sacco, Carla Lintas, Barbara Manzi, Paolo Curatolo, Maria Nobile, Catia Rigoletto, Massimo Molteni, Antonio M. Persico

**Affiliations:** 1 Unit of Child & Adolescent Neuropsychiatry, University Campus Bio-Medico, I-00128 Rome, Italy; ipiras@tgen.org (I.S.P.); stefano.gabrile@gmail.com (S.G.); laura.altieri.a@gmail.com (L.A.); federica.lombardi76@gmail.com (F.L.); r.sacco@unicampus.it (R.S.); c.lintas@unicampus.it (C.L.); 2 Neurogenomics Division, The Translational Genomics Research Institute, Phoenix, AZ 85254, USA; 3 Unit of Child and Adolescent Neuropsychiatry, University of Rome “Tor Vergata”, I-00133 Rome, Italy; bmanzi@libero.it (B.M.); curatolo@uniroma2.it (P.C.); 4 Child Psychopathology Unit, Scientific Institute, IRCCS ‘E. Medea’, I-23842 Bosisio Parini (LC), Italy; maria.nobile4@gmail.com (M.N.); Catia.rigoletto@bp.lnf.it (C.R.); massimo.molteni@lanostrafamiglia.it (M.M.); 5 Interdepartmental Program “Autism 0–90”, “G. Martino” University Hospital, University of Messina, I-98122 Messina, Italy

**Keywords:** arylesterase, attention deficit/hyperactivity disorder (ADHD), autism, autism spectrum disorder, developmental language disorder, organophosphate, paroxonase, pesticide, specific language impairment

## Abstract

Organophosphate compounds (OPs) interfere with neurodevelopment and are neurotoxic for humans and animals. They are first biotransformed to the more toxic oxon form, and then hydrolyzed to specific metabolites by the enzyme paraoxonase/arylesterase, encoded by the gene *PON1* located on human chr. 7q21.3. In autism spectrum disorder (ASD) and in attention-deficit/hyperactivity disorder (ADHD), a correlation between OP exposure and disease onset has been reported. In this case-control study, we aimed to replicate our previous work showing reduced levels of serum PON1 arylesterase activity in Italian and Caucasian-American ASD samples, and to extend our analysis to other neurodevelopmental disorders, namely ADHD and developmental language disorder (DLD), also known as specific language impairment (SLI). The arylesterase activity, measured using standard spectrophotometric methods, is significantly reduced in the ADHD, and not in the ASD sample compared with the controls. Our previous results seemingly stem from spuriously high arylesterase levels in the former control sample. Finally, genotyping SNPs rs705379 and rs662 using TDI-FP, a significant effect of rs705379 alleles on the serum arylesterase activity is observed in all of the subgroups tested, regardless of diagnosis, as well as a lack of association between *PON1* gene polymorphisms and ASD/ADHD susceptibility in the Italian population. In summary, the serum arylesterase activity is reduced in children and adolescents with ADHD, and this reduction is not due to the functional *PON1* gene variants assessed in this study. Based on previous literature, it may more likely reflect enhanced oxidative stress than specific genetic underpinnings.

## 1. Introduction

Organophosphate compounds (OPs) are widely employed as pesticides in agriculture and as insecticides for residential use. OPs do not act directly on living organisms, they are first biotransformed in the liver by cytochrome P-450-dependent monooxygenases to the more toxic oxon form, and then hydrolyzed to specific metabolites by the enzyme paraoxonase 1 [1]. Oxons are active as acetylcholinesterase (AChE) inhibitors, increasing the amounts and duration of the acetylcholine activity in the extracellular fluids. At low concentrations, oxons exert an effect on muscarinic M2 receptors, on cAMP response element-binding protein (CREB) phosphorylation [2], on the serotoninergic system [3], and are able to inhibit the expression of fibroblast growth factors [4]. More broadly, OPs can interfere with neurodevelopmental processes, including neuronal proliferation, differentiation, neurite outgrowth, synaptogenesis, apoptosis, and glial proliferation and differentiation [5]. Experimental data suggest that OPs may exert these actions either by interacting directly with the neuronal and glial cytoskeleton, or by targeting the cell signaling pathways regulating the phosphorylation of cytoskeletal proteins [6].

Through these multiple mechanisms, OPs yield important neurotoxic consequences in humans and animals, resulting in altered neurodevelopment [7,8,9]. Exposure in humans can occur in multiple contexts, through diet, residential proximity to fields sprayed with pesticides, home use of insecticides by exterminators or home owners, or occupational exposure of workers or their items [10]. The association of OP exposure with neurodevelopmental abnormalities has been extensively studied in the past few years. Muñoz-Quezada et al. (2013) reviewed a total of 27 studies, and only one study did not report data supporting the involvement of OP in neurodevelopment [10]. For autism spectrum disorder (ASD), the CHARGE study identified a positive association with prenatal residential proximity to OP pesticides in the second and third trimesters of pregnancy [11], supporting previous results demonstrating associations of ASD and pervasive developmental disorder (PDD) with gestational exposure to OP [12,13]. On the contrary, the urinary concentrations of several organophosphate metabolites derived from prenatal insecticide exposure were not correlated with children’s socio-communicative skills and with stereotypic behaviors assessed at 8 years of age [14].

Environmental factors can produce a variety of neurodevelopmental disorders, depending on genetic liability and the developmental timing of exposure [15]. In reference to OPs, the strongest evidence supports OP exposure as contributing to reduced cognitive performance [16]. However, as the diagnostic outcome is non-specific, contributions by OPs to neurodevelopmental disorders other than intellectual disability or ASD have been investigated. The possible contribution of OP exposure to attention-deficit/hyperactivity disorder (ADHD) has been addressed in several studies. A significant association has been reported between prenatal chlorpyrifos exposure and several neurodevelopmental disorders, including ADHD, delayed psychomotor development, and pervasive developmental disorders assessed at 3 years of age [17]. Bouchard et al. (2010) detected high levels of urinary dialkyl phosphate (DAP) metabolites derived from OP pesticides in ADHD children from 8 to 15 years of age [18]. In the CHAMACOS study, prenatal maternal urinary levels of DAP metabolites were associated with maternal reports of attention problems and ADHD assessed in the offspring at 5 years of age using the Conners Kiddie Continuous Performance Test (K-CPT ADHD) and a composite ADHD measure [19].

The enzyme Paraoxonase 1, encoded by the *PON1* gene located in human chr. 7q11.3 (OMIM: #168820), hydrolyzes the oxons, blunting the toxic effects of OPs [1,20]. PON1 is a polypeptide of 355 aminoacids primarily synthetized in the liver and secreted in the bloodstream, where it is found to be associated with high density lipoproteins (HDLs). It possesses different hydrolase activities, grouped into the following three classes, arylesterase, phosphotriesterase, and lactonase [21], displaying remarkable inter-individual differences in enzyme activity and serum concentration [22]. Reduced PON1 enzymatic activity, resulting from low-activity *PON1* genetic variants, increases toxic levels of OP metabolites and their persistence in the human body, therefore raising the probability of detrimental effects on CNS development, especially during pregnancy and early childhood. Three polymorphisms located in the *PON1* coding region play a key role in modulating the enzyme function and activity, namely, (a) rs662 (Q192R), influences enzymatic activity, namely the phosphotriesterase catalytic efficiency (i.e., paraoxonase) [23], whereas (b) PON1 enzyme amounts are determined by rs854560 (L55M), which influences protein stability [24,25,26,27], and (c) by rs705379 (C-108T) located in the promoter region, which influences transcription rates [28,29,30]. A recent metanalysis supports genetic contributions to neurodevelopmental disorders primarily from SNPs rs705379 (C-108T) and rs662 (Q192 R) [31].

Several studies have investigated the association between neurodevelopmental disorders and PON1 enzyme activity and/or gene polymorphisms. A significant association between the less active *PON1* variants, especially the R108 allele at SNP rs662, and ASD was detected in Caucasian-American families, but not in Italian families, pointing toward geographical specificities in gene–environment interactions [32]. Paşca et al. (2006, 2010) reported low levels of arylesterase activity in ASD patients vs. healthy controls [33,34]. We previously reported decreased serum arylesterase activity levels in autistic children compared with their first-degree relatives and matched controls, both in Italian and in Caucasian-American families [35]. In the same study, a significant effect of *PON1* SNPs rs705379 and rs662 on the serum arylesterase activity levels was detected in the ASD sample, whereas only rs705379 was influential in the controls and first-degree relatives [35]. Based on these converging results, decreased serum arylesterase activity has been considered as a putative familial marker for ASD. Reduced serum arylesterase and paraoxonase activities, as part of a broader increase in oxidative stress present in ADHD children and adults, was reported by most [36,37,38], although not all, studies [39]. Importantly, enhanced oxidative stress and reduced serum PON1 activity appear to normalize after three months of methylphenidate treatment [40]. To our knowledge, *PON1* gene polymorphisms in ADHD have not been investigated. Moreover, no study has focused on specific language impairment (SLI), nor is there sufficient evidence of connections between prenatal OP exposure and language development [41].

Based on these observations, the aim of the present study is to replicate and extend our previous results [35], verifying the sensitivity of reduced serum arylesterase activity as an ASD biomarker in a newly recruited sample of Italian autism cases, and its specificity, by investigating, in parallel, two distinct samples of cases with other neurodevelopmental disorders, namely ADHD and SLI. Measurements of the serum arylesterase activity are always paired with parallel genotyping of the C-108T and Q192R polymorphisms in the same patient and control samples.

## 2. Materials and Methods

### 2.1. Subjects

This case-control study involves 233 individuals of Italian descent, whose demographic characteristics are summarized in Table 1. Patients were recruited across the years 2009–2014, based on a clinical diagnosis of ASD, ADHD, or SLI, according to Diagnostic and Statistical Manual of Mental Disorders IV (DSM-IV) criteria [42]; patients were excluded if affected by a known or suspected genetic syndrome, acute or chronic inflammatory disease, focal or generalized neurological signs, MRI positive for brain malformations, or frequent seizures (i.e., >1 every 6 months). The ASD sample encompasses 28 ASD children, 23 fathers, and 26 mothers, belonging to 28 families (25 simplex and 3 multiplex). Thirty ADHD patients were collected at the University of Rome “Tor Vergata” and 52 children with DLD/SLI were recruited at IRCCS “La Nostra Famiglia” (Bosisio Parini, LC; prot. n. 79/09). All parents gave informed written consent for themselves and for their children. The consent form was approved by the Institutional Review Board of University ‘‘Campus Bio-Medico’’ (Rome, Italy; prot. n. 14.CI(98).2010). Healthy controls (N = 74) were recruited among blood donors, drawn at the Transfusion Medicine Center of U.C.B.M. (Rome, Italy). Controls were not age- and sex-matched to patients, but the age of the controls now overlapped with the age of ASD parents and was not as distant from the age of the children/adolescents with ADHD, ASD, or SLI as in our previous study [35]. Blood donors gave written consent for the use of their samples for research purposes.

### 2.2. Measurements of Serum Arylesterase Activity

The arylesterase activity was measured in the entire sample using standard spectrophotometric procedures [22,43,44], as described in detail in the Supplementary Methods.

Briefly, 1 μL of a 1:50 dilution of serum was added to 500 μL of a substrate solution (3.26 mM phenylacetate in 9 mM Tris-HCl pH 8.0 and 0.9 mM CaCl2). Then, the rate of hydrolysis was monitored in a 1 cm quartz cuvette by spectrophotometry using a SAFAS Monaco UV mc2 apparatus, measuring at 270 nm for two min at room temperature. The enzymatic activity was then quantified in Units/Liter, using molar extinction coefficients of 1.310 mol L^−1^ cm^−1^ for phenol. The serum volumes and dilutions were chosen to maintain the kinetics below 0.4 ∆OD/min, and the starting absorbance was kept as low as possible (i.e., <0.2). The arylesterase activities reported in this study represent the mean of 2–3 independent measurements, as each serum sample was independently diluted and measured twice, and a third measurement was performed if the initial results differed by more than 20% of the lower value. Validation was implemented by frequent blanking (negative control) and by measuring the same test sample at least three times a day for consistency (positive control).

### 2.3. Genotyping

Genomic DNA was isolated from EDTA-anticoagulated whole blood using the salting out method [45]. SNPs rs705379 and rs662 were genotyped in all of the samples using (1) PCR amplification and restriction digest or (2) by template-directed dye-terminator incorporation with fluorescence polarization (TDI-FP), as previously described [32,35]. The detailed methods are explained in the Supplementary Methods.

### 2.4. Statistical Analysis

The arylesterase levels were sqrt-transformed in order to achieve a normal distribution. The mean values among the samples were compared using one-way ANOVA followed by Tukey honest significance test (HSD) for pairwise comparisons. The alleles, genotype frequencies, Hardy–Weinberg equilibrium, and Fisher’s exact test for samples’ differentiation for SNPs rs705379 and rs662 were estimated using Arlequin 3.5 software using Markov Chain model with 10,000 steps [46]. Finally, correlation among arylesterase levels (dependent variable) and genotypes for SNPs rs705379 and rs662 (independent variables) were analyzed using a multiple regression model, setting up age and gender as covariates. ANOVA, HSD, and multiple regression analysis were executed using the SPSS v25 package.

## 3. Results

### 3.1. Arylesterase Activity

**Figure 1:**
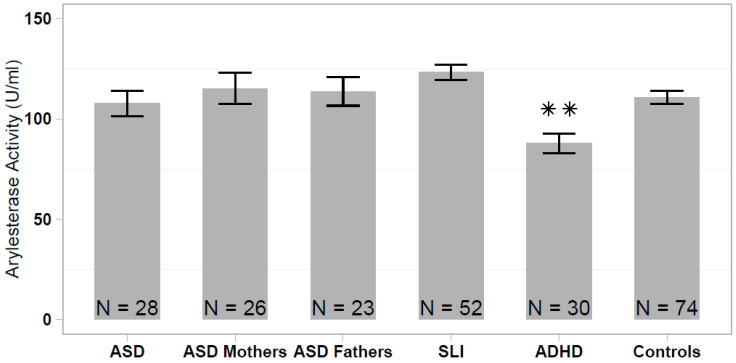
Serum arylesterase activity levels in patients with autism spectrum disorder (ASD), ASD patients’ parents, specific language impairment (SLI), attention deficit/hyperactivity disorder (ADHD), and healthy controls. Data are reported as mean ± standard error of the mean, and significant pairwise comparison between each patient/parent sample and healthy controls are displayed (** *p* < 0.01).

The serum arylesterase activity differed significantly among the analyzed samples (one-way ANOVA: F = 5.339; df = 5, 232; *p* < 0.001), ranging from 87.8 ± 27.0 U/mL (ADHD sample) to 123.3 ± 27.5 U/mL (SLI sample). The arylesterase activity was significantly lower in the ADHD sample compared with the healthy controls (HSD Tukey: *p* < 0.01), to SLI cases (*p* < 1.2 × 10^−5^) and to both ASD parent samples (*p* < 0.05), whereas differences between ADHD and ASD did not reach statistical significance (*p* = 0.136). No significant difference in the serum arylesterase activity was detected among the remaining non-ADHD samples (ASD vs SLI vs ASD parents vs. controls; Figure 1).

**Figure 2:**
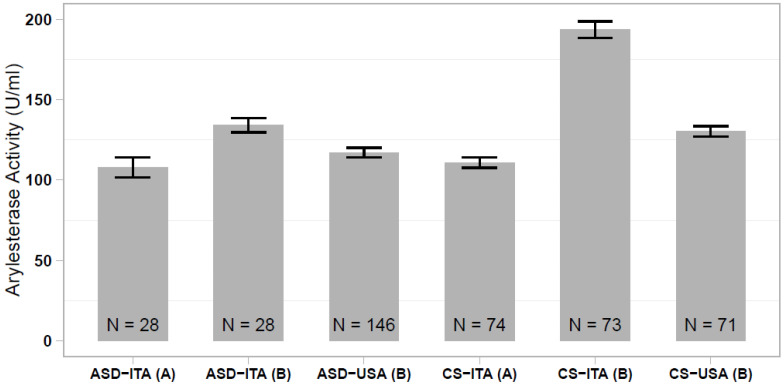
Comparison of serum arylesterase activity levels measured in ASD and control samples of Italian and Caucasian-American ethnicity, assessed (**A**) in the present work and (**B**) in the work of Gaita et al. (2010) [35].

In our previous study, we reported a significantly lower serum arylesterase activity in Italian and Caucasian-American ASD cases compared with ethnically-matched controls, although this case-control difference was much more prominent in the Italian sample [35]. Comparing all of the different case and control groups analyzed here and in the Gaita et al. (2010) study [35], we observed that the Italian controls assessed in the previous study stand out, displaying elevated arylesterase activity levels that differ largely from the present control sample (*p* < 1 × 10^−23^; Figure 2). We then investigated whether discordant results between the two studies could be due to an excess of hyperkinetic children in the Italian ASD sample assessed in our previous work. In the present sample of 28 ASD children, we found no difference in the serum arylesterase activity between 15 ASD and ADHD vs. 13 ASD only children (136.6 ± 6.1 vs. 134.5 ± 6.2 U/mL, *p* = 0.813). Hence, our previous results were likely due to the spuriously elevated serum arylesterase activity in the Italian control sample, rather than to a truly decreased enzymatic activity in the Italian ASD patient sample.

### 3.2. PON1 Genotypes

The genotype frequencies for rs705379 (C-108T) and rs662 (Q192R) in each sample group are reported in Table 2. Both SNPs were in Hardy–Weinberg equilibrium in each group after correction for multiple testing (significance level: *p* < 0.025), with the exception of a weak nominal disequilibrium for ASD mothers (*p* = 0.044). The genotype and allele frequencies are reported in Table 2, while odd ratios (O.R.) and P-values are presented in Supplementary Table S1. No global or pairwise significant difference between patients (ASD, ADHD, and SLI) vs. controls was detected for the genotype and allele distributions (Table 2, Supplementary Table S1), providing little evidence of susceptibility being conferred by these genetic variants for ASD, ADHD, and SLI in these Italian samples.

### 3.3. Correlation between Arylesterase Activity and PON1 Genotypes

**Figure 3:**
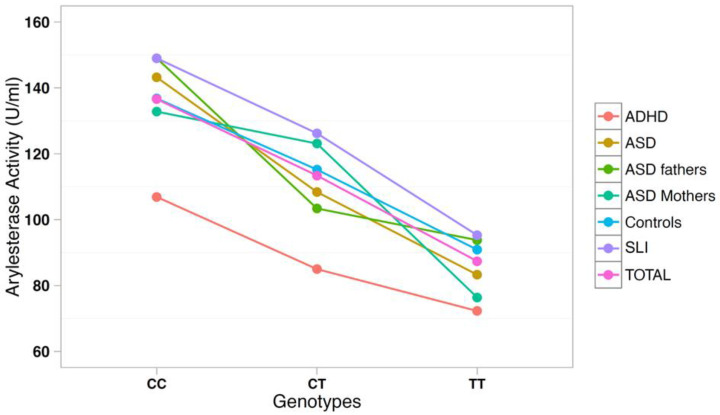
Arylesterase activity of the *PON1* genotype at SNP rs705379 (C-108T).

**Figure 4:**
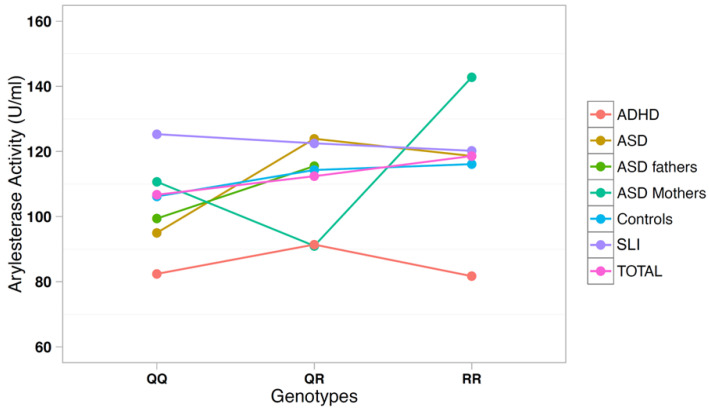
Arylesterase activity of the *PON1* genotype at SNP rs662 (Q192R).

The correlation between arylesterase activity and genotypes was investigated using a multiple regression model including age, gender, and diagnostic status as covariates (Table 3). SNP rs705379 influenced the arylesterase activity in the total sample (*p* = 6.9 × 10^−18^) and in each diagnostic subgroup, with a decreased arylesterase activity associated with the T allele in all neurodevelopmental disorders (Figure 3). On the other hand, SNP rs662 did not influence the arylesterase activity in the total sample (*p* = 0.240), nor in any neurodevelopmental disorder analyzed separately (Figure 4).

## 4. Discussion

In this work, we measured the *PON1* arylesterase activity in newly-recruited samples of patients diagnosed with different neurodevelopmental disorders, namely ASD, ADHD, and SLI, as well as healthy controls. Our study was designed with two main purposes, namely: (A) to replicate in an independent sample the strong association observed in our previous work between reduced serum arylesterase activity and ASD [35], and (B) to extend our investigation of *PON1* roles to other neurodevelopmental disorders. These results were intended to ultimately determine the sensitivity and specificity of reduced arylesterase activity as a biomarker of ASD. Instead, the most noteworthy result here is the presence of significantly decreased arylesterase activity in children with ADHD, and not with ASD, compared with healthy controls, to children with SLI, to both mothers and fathers of ASD children, while differences between ADHD and ASD children did not reach significance. A similar association was reported in a previous study involving an adult sample of ADHD cases from Turkey [38], who also displayed high levels of malondialdehyde, a marker of lipid peroxidation previously correlated with reduced serum arylesterase activity [47]. Similar changes in a variety of parameters associated with increased oxidative stress, including decreased serum arylesterase and paraoxonase activities, were reported in children and adolescents by most [36,37], though not all, studies [39]. Others reported enhanced oxidative stress affecting some parameters, but not arylesterase [40]. Our present results provide evidence of serum arylesterase activity being reduced to a significant extent in ADHD, pointing toward the need to further investigate enhanced oxidative stress as a plausible cause of this reduction in hyperactive children.

*PON1* allele and genotype frequencies at SNPs rs705379 and rs662 do not differ between ADHD patients and controls (Table 2). As expected, rs705379 is significantly associated with arylesterase activity in the ADHD sample according to the pattern CC > CT > TT, while no association with rs662 is found. These results are predictable, because the T allele at rs705379 reduces the *PON1* gene expression in all samples to an equal extent (Table 3 and Figure 3), and no association between rs662 and ADHD has been reported to date. Although our samples are too small to allow for meaningful case-control genetic association analyses, genotype and allele distributions are so overlapping that the decreased arylesterase activity in our ADHD children is unlikely to stem from genetic differences, at least at these two functional SNPs, but may more likely reflect co-variance of hyperactivity and oxidative stress, which has been reported to normalize with methylphenidate treatment [40].

With respect to ASD, the significant decrease in the serum arylesterase activity we previously reported contrasting Italian ASD cases vs. healthy controls is not replicated here [35]. Italian controls from our previous study indeed show prominently elevated arylesterase activity compared not only to the Italian controls enrolled in the present study, but also to the Caucasian-American controls assessed previously [35]. We thus believe that an erroneous sampling scheme of our previous Italian control sample may have, by contrast, generated a spurious “decrease” in the Italian ASD sample, which seemingly represents a false-positive finding. In the same study, we detected a statistically significant, but less sizable decrease in the serum arylesterase activity in the Caucasian-American sample of ASD patients compared with Caucasian-American controls [35]. The extent of this decrease was comparable with data from Paşca et al. (2006 and 2010), reporting a significantly lower arylesterase activity in ASD patients vs. healthy controls in two independent samples from Romania [33,34]. No difference in arylesterase activity between Italian autistic and control individuals was also reported by Hayek et al. (2017), who instead detected a significantly lower lactonase activity in their ASD sample [48]. In the present study, we recruited controls among blood donors, aiming to obtain samples from perfectly healthy volunteers. In our initial study, controls had been recruited among individuals whose blood was drawn at the Laboratory of the Campus Bio-Medico University Hospital (Rome, Italy), as prescribed by family practitioners for a routine physical check-up [35]. Only individuals with normal lab test results were recruited, yet some bias may have been introduced by this sampling strategy, coupled with the older mean age of those controls (54.5 ± 17.4 vs 33.8 ± 10.8 in the present control group). In addition, different ADHD comorbidity rates could represent another source of bias in comparing serum PON1 activity among distinct ASD samples, on top of the usual interindividual variability in clinical presentation. However, we did not detect any major difference in the serum arylesterase activity correlated with the presence/absence of co-morbid ADHD. Larger and well-designed studies will be necessary to investigate the relationship between hyperactivity, sensory self-stimulation, and intense stereotypic behaviors on the one hand, and enhanced oxidative stress with reduced serum PON1 activity on the other.

These genetic analyses confirm our previous report of a lack of association between *PON1* gene polymorphisms and ASD in case-control samples of Italian descent [32]. Genotype–phenotype correlations also confirm the consistent influence of genotypes and alleles at SNP rs705379 (C-108T) on the serum arylesterase activity, regardless of diagnostic status, as previously reported [35]. Furthermore, we once again did not find the R allele at SNP rs662 to be significantly associated with decreased arylesterase activity in Italian ASD cases and first-degree-relatives, as it occurred in the Caucasian-American samples [32]. A similar lack of association between rs662 and serum arylesterase activity in ASD was also reported by Paşca et al. (2010) in Romanian samples [34].

The main limitation of the present study is that it is focused exclusively on arylesterase activity and PON1 genetics, in line with our previous contribution [35], and not on oxidative stress as a whole. Consequently, no other parameters of oxidative stress were measured. Based on our data, we can affirm that the arylesterase activity is decreased in ADHD, and that this decrease is not due to the functional genetic variants assessed here. Our interpretation linking reduced serum arylesterase activity to increased oxidative stress is based exclusively on previous studies [36,37,38,40], and must be viewed with caution, until we can assess PON1 enzymatic activities in parallel with other biomarkers of oxidative stress in a newly recruited sample of patients and controls. Secondly, patients were subgrouped according to their primary diagnosis, and their comorbidities were not considered in this study. This is due to DSM-IV criteria not allowing for the recognition of ADHD as an independent, co-morbid disorder in the presence of an ASD diagnosis, as well as language impairment being frequently associated to ASD, despite “pure” SLI being a separate disorder. Neurodevelopmental disorders often represent only partly different and frequently co-morbid expressions of the same underlying genetic or epigenetic trigger. Despite this frequent overlap, which to some extent limits the validity of the sampling distinctions made in this study, the primary diagnosis retains its validity, because it almost always represents the main cause of clinical concern. Lastly, the sample sizes in this study are small, especially for genetic association analyses. Nonetheless, the influence exerted by SNP rs705379 (C-108T) on the serum arylesterase activity reaches statistical significance in all diagnostic subgroups, supporting the reliability and validity of our observation.

## 5. Conclusions

This study reports on the presence of significantly reduced serum arylesterase activity in children with ADHD. Our results disconfirm the apparent decrease we initially observed in a sample of Italian autistic children, seemingly due to the spuriously elevated arylesterase activity in the control sample assessed previously [35]. Conceivably, decreased arylesterase activity in ADHD may represent either a “state” condition, possibly linked to enhanced oxidative stress in hyperactive children [36,37,38], or a “trait” condition conferring vulnerability to neurodevelopmental issues, but not derived from the functional *PON1* SNPs assessed in this study. Moreover, in ASD, there may still be a modest decrease in arylesterase activity, not in Italians [46], but possibly in other ethnic groups, such as Caucasian-Americans [35] and Romanians [33,34]. This outcome could reconcile biochemical results with genetic data, supporting an association between *PON1* alleles and ASD in North America but not in Italy [32]. Collectively, the present results tend to exclude that the serum arylesterase activity may serve as a clinically useful biomarker for ASD, because even assuming the latter scenario as correct, there seems to be too much overlap between autistic cases and controls to ensure a clinically useful degree of sensitivity (Figure 2, right panel) [35]. We cannot exclude that other PON1 enzymatic activities, such as lactonase, may distinguish ASD cases and controls with sufficient sensitivity [48].

From a genetic standpoint, *PON1* SNP rs705379 confirms its influence on the serum arylesterase activity, not only in ADHD, but in all diagnostic groups to an equal extent. Data regarding the role of SNP rs662 are a lot less consistent in different studies. This may be due to relatively small sample sizes, but it may also reflect geographical region-specific gene–environment interactions [32]. Indeed, different patterns of use of different OPs for different purposes in different geographical areas may place differential selective pressure on ASD development. In this regard, it may be important to consider that environmental factors negatively affecting neurodevelopment yield clinical consequences that are non-specific in terms of clinical diagnoses [15], and this may add another layer of complexity when trying to replicate previous findings starting from patient samples recruited by clinical diagnosis and not according to a broader “neurodevelopmental” dimension.

## Figures and Tables

**Table 1:** Demographic characteristic of all of the analyzed samples (total N = 233 individuals).

Sample		Age		Gender		
Groups	N	Mean ± SD	Range	Males	Females	M/F Ratio
ASD patients	28	8.5 ± 5.1	3–20	23 (82.1%)	5 (17.9%)	4.6:1
ADHD patients	30	11.8 ± 3.6	6–19	28 (93.3%)	2 (6.7%)	14:1
SLI patients	52	7.4 ± 3.2	2–13	29 (55.8%)	23 (44.2%)	1.3:1
Fathers ASD	23	46.1 ± 10.6	32–81	23 (100.0%)	−	−
Mothers ASD	26	41.8 ± 7.8	28–60	−	26 (100.0%)	−
Controls	74	33.8 ± 10.8	18–57	39 (52.7%)	35 (47.3)	1.1:1

**Table 2:** Allele and genotype distribution of SNPs rs705379 (C-108T) and rs662 (Q192R) in the different samples analyzed. Data are presented as N (%).

SNP	Genotype/Allele	ASD (N = 28)	ADHD (N = 28)	SLI (N = 52)	ASD Mothers (N = 26)	ASD Fathers (N = 23)	Controls (N = 74)	Total (N = 232)
rs705379	CC	3 (10.7)	7 (25.0)	11 (21.1)	5 (19.2)	5 (21.7)	12 (16.2)	43 (18.5)
	CT	20 (71.4)	12 (42.9)	28 (53.8)	14 (53.8)	12 (52.1)	38 (51.3)	125 (53.9)
	TT	5 (17.9)	9 (32.1)	13 (25)	7 (26.9)	6 (26.1)	24 (32.4)	64 (27.6)
	C	26 (46.4)	26 (46.4)	50 (48.1)	24 (46.2)	22 (47.8)	62 (41.9)	211 (45.5)
	T	30 (53.6)	30 (53.6)	54 (51.9)	28 (53.8)	24 (52.2)	86 (58.1)	253 (54.5)
rs662	QQ	15 (53.6)	9 (32.1)	22 (42.3)	16 (61.5)	8 (34.8)	33 (44.6)	104 (44.8)
	QR	9 (32.1)	15 (53.6)	22 (42.3)	6 (23.1)	14 (60.9)	35 (47.3)	101 (43.5)
	RR	4 (14.3)	4 (14.3)	8 (15.4)	4 (15.4)	1 (4.3)	6 (8.1)	27 (11.6)
	Q	39 (69.6)	33 (58.9)	66 (63.5)	38 (73.1)	30 (65.2)	101 (68.2)	309 (66.6)
	R	17 (30.4)	23 (41.1)	38 (36.5)	14 (26.9)	16 (34.8)	47 (31.8)	155 (33.4)

**Table 3:** Arylesterase activity levels in each sample for SNPs rs705379 (C-108T) and rs662 (Q192R).

SNP	Genotype	ASD (N = 28)	ADHD (N = 28)	SLI (N = 52)	ASD Mothers (N = 26)	ASD Fathers (N = 23)	Controls (N = 74)	Total (N = 232)
rs705379 (C108T)	CC	143.2	106.9	149	132.8	149	136.8	136.6
	CT	108.4	85	126.2	123.1	103.4	115.2	113.4
	TT	83.3	72.3	95.3	76.4	93.8	90.9	87.4
*p*-value		9.0 × 10^−3^	0.021	1.9 × 10^−7^	2.8 × 10^−3^	0.010	1.9 × 10^−7^	6.9 × 10^−18^
rs662 (Q192R)	QQ	95	82.4	125.3	110.7	99.4	106.2	106.7
	QR	123.9	91.4	122.5	91	115.5	114.3	112.4
	RR	118.6	81.7	120.2	142.8	170.3	116.1	118.6
*p*-value		0.119	0.627	0.418	0.217	0.073	0.692	0.241

## Data Availability

The data presented in this study are available on request from the corresponding author.

## Supplementary Materials

Supplementary Methods are available online at https://www.mdpi.com/2076-3921/10/2/164/s1.
