# Supplementary material for: Reevaluation of Serum Arylesterase Activity in Neurodevelopmental Disorders
Source: Antioxidants (Basel). 2021 Jan 22;10(2):164. doi: 10.3390/antiox10020164 (PMC7912005; doi:10.3390/antiox10020164)
Supplement: Supplementary file 1 [file antioxidants-10-00164-s001.pdf]

SUPPLEMENTARY MATERIAL

**Reevaluation of serum arylesterase activity in neurodevelopmental disorders**

**Ignazio Stefano Piras [1,2], Stefano Gabriele [1], Laura Altieri [1], Federica Lombardi [1], Roberto Sacco [1], Carla Lintas [1], Barbara Manzi [3], Paolo Curatolo [3], Maria Nobile [4], Catia Rigoletto [4], Massimo Molteni [4], and Antonio M. Persico [4]****

1 Unit of Child & Adolescent Neuropsychiatry, University Campus Bio-Medico, I-00128 Rome, Italy; ipiras@tgen.org (I.S.P.); stefano.gabrile@gmail.com (S.G.); laura.altieri.a@gmail.com (L.A.); federica.lombardi76@gmail.com (F.L.); r.sacco@unicampus.it (R.S.); c.lintas@unicampus.it (C.L.)
2 Neurogenomics Division, The Translational Genomics Research Institute, Phoenix, AZ 85254, USA; ipiras@tgen.org
3 Unit of Child and Adolescent Neuropsychiatry, University of Rome "Tor Vergata", I-00133 Rome, Italy; bmanzi@libero.it (B.M.); curatolo@uniroma2.it (P.C.)
4 Child Psychopathology Unit, Scientific Institute, IRCCS 'E. Medea', I-23842 Bosisio Parini (LC), Italy; maria.nobile4@gmail.com (M.N.); Catia.rigoletto@bp.lnf.it (C.R.); massimo.molteni@lanostrafamiglia.it (M.M.)
5 Interdepartmental Program "Autism 0–90", "G. Martino" University Hospital, University of Messina,
I-98122 Messina, Italy
* Correspondence: apersico@unime.it

SUPPLEMENTARY METHODS

*Measurement of serum arylesterase[1,2] and diazoxonase[3] enzymatic activities*

To measure arylesterase, 1 ◎l of a 1:50 dilution of serum was added to 500 ◎l of substrate solution (3.26 mM phenylacetate in 9 mM Tris-HCl pH 8.0 and 0.9 mM $CaCl_2$). Diazoxonase activity was measured by mixing in borosilicate glass tubes 2,5 ◎L of undiluted serum with 500 ◎l of substrate solution containing 0,5 mM diazoxon in 0.1 M Tris-HCl pH 8.5, 2.0 mM $CaCl_2$, and 2.0 M NaCl. For both activities, the rate of hydrolysis was monitored in a 1-cm quartz cuvette by spectrophotometry using a SAFAS Monaco UV mc2 apparatus, measuring at 270 nm for two minutes at room temperature. Enzymatic activity was then quantified in Units/Liter, using molar extinction coefficients of 1,310 and 3,030 mol $L^{-1}$ $cm^{-1}$ for phenol and diazoxon, respectively. Serum volumes and dilutions were chosen to maintain the kinetics below 0.4 ΔOD/min and starting absorbance was kept

as low as possible (i.e., <0.2). Arylesterase and diazoxonase activities reported in this study represent the mean of 2-to-3 independent measurements, as each serum sample was independently diluted and measured twice, and a third measurement was performed if initial results differed by more than 20% of the lower value. Validation was implemented by frequent blanking (negative control) and by measuring the same test sample at least three times a day for consistency (positive control).

*Genotyping*

*1) PCR amplification and restriction digest*:[4] The *PON1* SNP rs705379 (C-108T) was amplified using primers GACCGCAAGCCACGCCTTCTGTGCACC and TGCAGCCGCAGCCCTGCTGGGGCAGCGCCGATTGGCCCGCCGC with 5% DMSO and an annealing temperature of 63°C for 35 cycles. The 109 bp fragment was digested with *Bst*UI, yielding 67 and 42 bp fragments in the presence of the C allele; (b) rs662 (Q192R) was amplified using primers TATTGTTGCTGTGGGACCTGAG and CACGCTAAACCCAAATACATCTC at an annealing temperature of 60°C for 35 cycles. The 99 bp fragment was digested with *Alw*I, yielding 66 and 33 bp fragments with the R allele.

*2) Template-directed Dye-terminator Incorporation with Fluorescence Polarization (TDI-FP):* rs662 (Q192R) was genotyped also by TDI-FP, using the same primers as above for PCR amplification, and the following SNP primer for dye-terminator incorporation: TGATCACTATTTTCTTGACCCCTACTTAC. PCR reactions were set up using MULTIPLATE 96 well plates (MJ Research, Cat. MLP-9601), as follows:

| | |
|---|---|
| Water | 3,55 µl |
| 10x buffer | 0,62 µl |
| dNTPs (2,5 mM each) | 0,25 µl |
| Primer F (20 pM) | 0,25 µl |
| Primer R (20 pM) | 0,25µµl |
| Taq polimerase | 0,08 µl |
| DNA (50 ng) | 1,25 µl of a 40 ng/µl dilution |
| | |
| Total Volume | 6,25 µl |

PCR reactions were then run at 95°C for 3 min, followed by 35 cycles at 95°C for 1 min, 60°C for 1 min and 72°C for 1 min, followed by 72°C for 8 min. PCR clean-up was performed in black Hard-shell Thin-Wall microplates (MJ Research), modifying the manufacturer protocol (Perkin-Elmer) with the addition of exonuclease I (New England Biolabs, code #M0293S) and alkaline phosphatase (Sigma-Aldrich, code #P-9088), as follows:

| | |
|---|---|
| Water | 3,63◎◎l |
| PCR Clean-up dilution buffer | 1,65 ◎◎l |
| Exonuclease | 0,04 ◎◎l |
| Alkaline phosphatase | 0,33 ◎◎l |
| PCR Clean-up reagent 10X | 0,2 ◎◎l |
| PPase reagent | 0,15 ◎l |
| PCR reaction | 1 ◎l |
| Total volume | 7 ◎l |

Clean-up reactions were perforned at 37°C for 60 min, followed by enzyme inactivation at 80°C for 15 min. Finally, primer extension with the fluorescent dye-terminator was performed preparing the AcycloPrime-FP Mix according to the manufacturer's instructions, as follows:

| | |
|---|---|
| Water | 9,45◎◎l |
| 10X Reaction buffer | 2 ◎◎l |
| AcycloTerminator Mix | 1 ◎◎l |
| SNP primer (10 ◎M) | 0,5◎◎l |
| AcycloPol | 0,05 ◎◎l |
| Total volume | 13 ◎l |

A total of 13 ◎l of reaction mix was diluted to a final volume of 20 ◎l, placed on a thermal cycler and run at 95°C for 2 min, followed by a total of 20, 35, and 50 cycles at 95°C for 15 sec, followed by 55°C for 30 sec. After 20, 35, and 50 cycles, fluorescent polarization from R110 and TAMRA was read for 0.2 sec using a Victor2 plate reader, yielding QQ, QR, and RR genotypes. Negative and positive controls were included in each plate to ensure genotyping reliability.

*3) TaqMan® SNP Genotyping Assays:* SNP rs705379 was amplified using a PCR mixture containing approximately 500ng whole genome amplified DNA, iTAQ Supermix with ROX in a 1x concentration, TaqMan® SNP Genotyping Assay C__11708905_10 mix in a 1.2x concentration, and deionized water in a final volume of 10µl. Thermal-cycling conditions used were 95ºC for 3 minutes, followed by 55 cycles of 95ºC for 15 seconds and 62ºC for 45 seconds. SNP rs662 was amplified using a PCR mixture containing approximately 600ng whole genome amplified DNA, iTAQ Supermix with ROX (Biorad, Hercules, CA) in a 1x concentration, TaqMan® SNP Genotyping Assay C__2548962_20 mix in a 0.6x concentration, and deionized water in a final volume of 5µl. Thermal-cycling conditions were 95ºC for 3 minutes, followed by 50 cycles of 95ºC for 15 seconds and 60ºC for 45 seconds. Following PCR, an endpoint reading was done using the ABI Prism® 7000 Sequence Detection System.

*Quantitation of serum PON protein amounts by ELISA*

Serum PON protein concentrations were determined using an ELISA method, as previously described.[5,6] Briefly, all incubations were performed at room temperature with vigorous shaking, and wells were washed between all steps with phosphate buffer saline (PBS) containing 0.1% bovine serum albumin (BSA). Serum samples were diluted 1:4000 in 0.05M sodium carbonate buffer, added to a 96-well plate and incubated overnight. Subsequently, 1% BSA diluted in PBS was added as a blocking agent. Wells were then incubated for 1 hour with a rabbit anti-human *PON1* polyclonal antibody, obtained as previously described[5] and diluted 1:6400 with 1% BSA in PBS. Following washes, samples were incubated for 1 hour with a peroxidase-conjugated goat anti-rabbit IgG (Sigma-Aldrich, Saint Louis, USA). Finally, samples were exposed to the peroxidase substrate tetramethylbenzidine and its chromogenic reaction was stopped with 2M sulphuric acid. Plates were read at 450 nm using a Synergy microplate reader (BioTek Instruments Inc, Vermont, USA).

*References*


1. Kitchen, B.J., Masters, C.J., Winzor, D.J. Effects of lipid removal on the molecular size and kinetic properties of bovine plasma arylesterase. *Biochem J* **1973**, *135*, 93-99. DOI: 10.1042/bj1350093
2. Furlong, C.E., Richter, R.J., Seidel, S.L., Motulsky, A.G.. Role of genetic polymorphism of human plasma paraoxonase/arylesterase in hydrolysis of the insecticide metabolites chlorpyrifos oxon and paraoxon. *Am J Hum Genet* **1988**, *43*, 230-238.
3. Richter, R., Furlong, C.E. Determination of paraoxonase (PON1) status requires more than genotyping. *Pharmacogenetics* **1999**, *9*, 745-753.
4. D'Amelio, M., Ricci, I., Sacco, R., Liu, X., D'Agruma, L., Muscarella, L.A., Guarnieri, V., Militerni, R., Bravaccio, C., Elia, M., Schneider, C., Melmed, R., Trillo, S., Pascucci, T., Puglisi-Allegra, S., Reichelt, K.L., Macciardi, F., Holden, J.J., Persico, A.M. Paraoxonase gene variants are associated with autism in North America, but not in Italy: possible regional specificity in gene-environment interactions. *Mol Psychiatry* **2005**, *10*, 1006-1016. DOI: 10.1038/sj.mp.4001714
5. Marsillach, J., Mackness, B., Mackness, M., Riu, F., Beltrán, R., Joven, J., Camps, J. Immunohistochemical analysis of paraoxonases-1, 2, and 3 expression in normal mouse tissues. *Free Radic Biol Med* **2008**, *45*, 146-157. DOI: 10.1016/j.freeradbiomed.2008.03.023
6. Marsillach, J., Camps, J., Ferré, N., Beltrán, R., Rull, A., Mackness, B., Mackness, M., Joven, J., 2009. Paraoxonase-1 is related to inflammation, fibrosis and PPAR delta in experimental liver disease. *BMC Gastroenterol* **2009**, *9*, 3. DOI: 10.1186/1471-230X-9-3

***Supplementary Table S1. Odds Ratios (O.R.) and 95% confidence intervals with relative Z statistics and P values for PON1 genotypes and alleles at SNPs rs705379 (C-108T) and rs662 (Q192R) in the different samples analyzed.***

| **SNP** | **Genotype/Allele** | | **ASD (N = 28)** | **ADHD (N = 28)** | **SLI (N = 52)** | **ASD mothers (N = 26)** | **ASD fathers (N = 23)** |
|---|---|---|---|---|---|---|---|
| **rs705379** | CC | O.R. | 0.6200 | 1.7222 | 1.3862 | 1.2302 | 1.4352 |
| | | 95 % CI | 0.1611-2.3861 | 0.5994-4.9481 | 0.5589-3.4379 | 0.3877-3.9034 | 0.4464-4.6142 |
| | | z statistic | 0.695 | 1.010 | 0.705 | 0.352 | 0.606 |
| | | P-value | 0.4869 | 0.3127 | 0.4810 | 0.7251 | 0.5443 |
| | CT | O.R. | 2.3684 | 0.7105 | 1.1053 | 1.1053 | 1.0335 |
| | | 95 % CI | 0.9269-6.0518 | 0.2958-1.7068 | 0.5430-2.2496 | 0.4513-2.7069 | 0.4051-2.6367 |
| | | z statistic | 1.801 | 0.764 | 0.276 | 0.219 | 0.069 |
| | | P-value | 0.0716 | 0.4447 | 0.7825 | 0.8267 | 0.9450 |
| | TT | O.R. | 0.4529 | 0.9868 | 0.6944 | 0.7675 | 0.7353 |
| | | 95 % CI | 0.1534-1.3373 | 0.3891-2.5025 | 0.3138-1.5367 | 0.2841-2.0738 | 0.2572-2.1019 |
| | | z statistic | 1.434 | 0.028 | 0.900 | 0.522 | 0.574 |
| | | P-value | 0.1516 | 0.9777 | 0.3682 | 0.6019 | 0.5661 |
| | C/T | O.R. | 1.2022 | 1.2022 | 1.2843 | 1.1889 | 1.2715 |
| | | 95 % CI | 0.6477-2.2312 | 0.6477-2.2312 | 0.7754-2.1273 | 0.6297-2.2447 | 0.6543-2.4707 |
| | | z statistic | 0.584 | 0.584 | 0.972 | 0.534 | 0.709 |
| | | P-value | 0.5595 | 0.5595 | 0.3310 | 0.5935 | 0.4785 |
| **rs662** | QQ | O.R. | 1.4336 | 0.5885 | 0.9111 | 1.9879 | 0.6626 |
| | | 95 % CI | 0.5989-3.4313 | 0.2355-1.4709 | 0.4452-1.8645 | 0.7974-4.9555 | 0.2505-1.7529 |
| | | z statistic | 0.809 | 1.134 | 0.255 | 1.474 | 0.829 |

| | | | | | | |
|---|---|---|---|---|---|---|
| | P-value | 0.4186 | 0.2567 | 0.7989 | 0.1404 | 0.4070 |
| QR | O.R. | 0.5278 | 1.2857 | 0.8171 | 0.3343 | 1.733 |
| | 95 % CI | 0.2114-1.3179 | 0.5377-3.0741 | 0.3998-1.6701 | 0.1205-0.9271 | 0.6679-4.4984 |
| | z statistic | 1.369 | 0.565 | 0.554 | 2.105 | 1.130 |
| | P-value | 0.1711 | 0.5720 | 0.5798 | 0.0353 | 0.2583 |
| RR | O.R. | 1.8889 | 1.8889 | 2.0606 | 2.0606 | 0.5152 |
| | 95 % CI | 0.4906-7.2720 | 0.4906-7.2720 | 0.6694-6.3433 | 0.5324-7.9760 | 0.0588-4.5161 |
| | z statistic | 0.925 | 0.925 | 1.260 | 1.047 | 0.599 |
| | P-value | 0.3551 | 0.3551 | 0.2076 | 0.2951 | 0.5493 |
| Q/R | O.R. | 1.0676 | 0.6677 | 0.8082 | 1.2631 | 0.8725 |
| | 95 % CI | 0.5482-2.0790 | 0.3538-1.2599 | 0.4766-1.3708 | 0.6249-2.5531 | 0.4339-1.7544 |
| | z statistic | 0.192 | 1.247 | 0.790 | 0.650 | 0.383 |
| | P-value | 0.8475 | 0.2124 | 0.4296 | 0.5154 | 0.7020 |
